# Easy-to-Engineer Flexible Nanoelectrode Sensor from an Inexpensive Overhead Projector Sheet for Sweat Neuropeptide-Y Detection

**DOI:** 10.1021/acsabm.4c01229

**Published:** 2024-11-16

**Authors:** Jayakrishnan Aerathupalathu Janardhanan, Jia-Wei She, Hsiao-hua Yu

**Affiliations:** † Smart Organic Materials Laboratory, Institute of Chemistry, Academia Sinica, Taipei City 115201, Taiwan; ‡ Taiwan International Graduate Program (TIGP), Sustainable Chemical Science and Technology, Academia Sinica, Taipei City 115201, Taiwan; § Department of Applied Chemistry, National Yang Ming Chiao Tung University, Hsinchu 300, Taiwan; ∥ Taiwan International Graduate Program (TIGP), Nano Science and Technology Program, Department of Engineering and System Science, National Tsing Hua University, Hsinchu 300, Taiwan

**Keywords:** PEDOT-derived nanobiosensor, OHP nanoelectrode, functionalized PEDOT nanostructures, Neuropeptide-Y detection, sweat biomarkers, wearable electronics

## Abstract

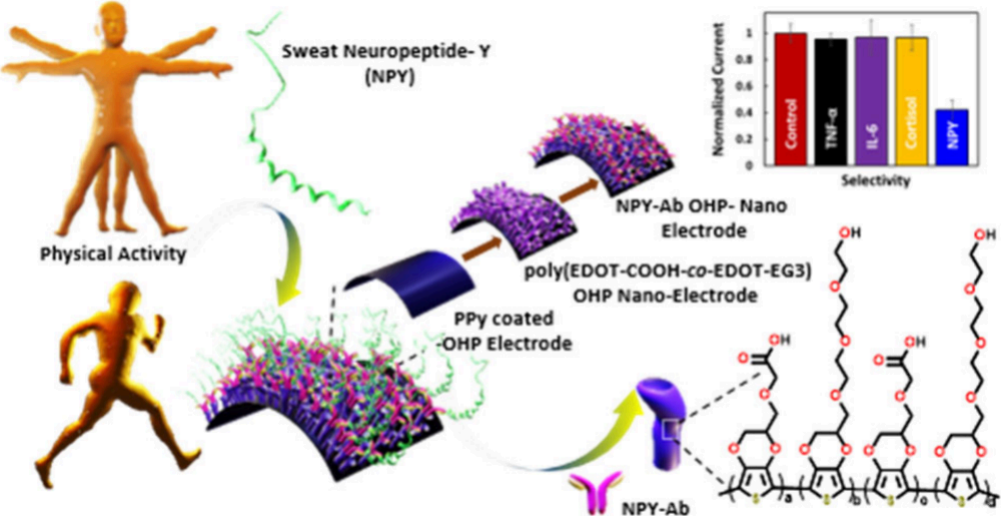

In this paper, we report an inexpensive and easy-to-engineer flexible nanobiosensor electrode platform by exploring a nonconductive overhead projector (OHP) sheet for sweat Neuropeptide-Y (NPY) detection, a potential biomarker for stress, cardiovascular regulation, appetite, etc. We converted a nonconductive OHP sheet into a conductive nanobiosensor electrode platform with a hybrid polymerization method, which consists of interfacial polymerization of pyrrole and a template-free electropolymerization technique to decorate the electrode platform with poly(EDOT-COOH-*co*-EDOT-EG3) nanotubes. The selection of poly(EDOT-COOH) features an easy conjugation of NPY antibody (NPY-Ab) through EDC/Sulfo-NHS coupling chemistry, while poly(EDOT-EG3) is best known to reduce nonspecific binding of biomolecules. The antibody conjugation on the polymer surface was characterized by a quartz crystal microbalance, Fourier transform infrared spectroscopy, X-ray photoelectron spectroscopy, and chronoamperometry techniques. The OHP nanosensor platform exhibited the successful detection of NPY analyte through a chronoamperometry method in phosphate-buffered saline with a wide range of concentrations from 1 pg/mL to 1 μg/mL with a limit of detection of 0.68 pg/mL having good linearity (*R*^2^ = 0.9841). The sensor platform exhibited excellent stability, reproducibility, repeatability, and a shelf-life of 13 days. Furthermore, the sensor showed superior selectivity to a 100 pg/mL NPY analyte among other interfering compounds such as tumor necrosis factor α, cortisol, and Interleukin-6. The clinical practicality of the sensor was confirmed through the detection of 100 pg/mL NPY spiked artificial perspiration, highlighting the possibility of integrating the sensor platform to wearable healthcare applications.

## Introduction

Accurate, precise, timely, and early diagnoses of serious diseases are essential for the advancement of a healthy community.^1−3^ Bacterial infections, influenza diseases, cancer metastasis, and metabolic and genetic disorders can lead to life-threatening situations if not detected in the initial stage.^4−6^ The surge of the Covid-19 pandemic and its catastrophic situations showed that advanced sensing tools for early disease diagnosis are highly demanded.^7^ The traditional clinical approach for disease diagnosis utilizes blood, urine, and serum samples. However, such diagnosis tools are hospital-oriented with the use of sophisticated and expensive instrumentation, extensive sample collection, requirement of a skilled person, time-consuming experimental processes, etc. Furthermore, some of the diagnosis methods are invasive, which might be uncomfortable for pain-sensitive patients when repeated sample extraction using a lancet puncture is required. Recent fostering of wearable electronics technology for the real-time monitoring of many diseases gives a promising solution to complex diagnostic procedures.^8^ Researchers have made significant breakthroughs in the development of wearable biosensors through the design and development of noninvasive sensor platforms.^9,10^ Such wearable electronics healthcare systems can switch complex diagnostic protocols from large room to small chip-based detection platforms. Therefore, the design and development of lab-on-a-chip biosensor platforms or lab-on-an-organ bioelectronics interfaces can serve as next-generation smart systems to continuously monitor personalized health status from home-setting platforms.^11^

Conducting polymer nanostructures/nanocomposites have proven to be one of the top-notch choices for designing novel biosensor platforms as well as bioelectronics interface materials.^12−15^ Among various conducting polymers, poly(3,4-ethylenedioxythiophene) (PEDOT) derivatives are attractive as versatile candidates because of their low oxidation potential, high biocompatibility, and ease of side-chain functionalization.^16−18^ In addition, the nanoscale engineering of such polymeric materials, in combination with molecular effects, can tune materials for applications in biomedical engineering, wearable sensing, drug delivery, electronics, energy storage, etc.^19−25^ Moreover, PEDOT and its derivatives were explored for the development of biosensors for the detection of glucose, lactate, cortisol, ammonia, ions, and other metabolites.^26−32^ The advantage of functionalized PEDOT materials highlights the conjugation of sensor probes such as antibodies, aptamers, or enzymes through polymer side-chain functionalization. In addition, fine-tuning of the polymer surface morphology at nanoscale dimensions can produce highly sensitive nanobiosensors.^25,33−36^ Furthermore, the high biocompatibility of PEDOT and its derivatives to design nanobiosensors offer their potential applications in advanced wearable electronics for biomarker detection.^37^

Among the other body fluids for biomarker detection, sweat-based biosensors are an attractive choice due to the continuous monitoring of diseases and easy as well as noninvasive sample collection and storage, without painful lancet puncture to extract the fluid. Recent research outcomes on the development of sweat-based biosensors highlight the design of promising platforms for the detection of important biomarkers such as glucose, lactate, cortisol, neuropeptides, sodium, potassium, calcium, urea, ammonia, and various cytokines.^38−42^ Among the various biomarkers and analytes presented in sweat, Neuropeptide-Y (NPY) is a particularly important biomarker that is involved in various homeostasis and physiological activities. NPY is a 36 amino acid neuropeptide produced by neurons in the central and peripheral nervous systems. NPY has an influence on cellular communications inside the body, food uptake, connections with obesity, cardiovascular control, circadian rhythm, stress, anxiety, and depression.^43−45^ Studies have shown that an abnormal concentration of NPY in the body can lead to major diseases and disorders, particularly stress-related health problems.^46,47^ Therefore, the quantification of NPY in body fluids can pass on the information about an individual’s stress level. The normal concentration of NPY in sweat ranges from 50 to 200 pg/mL. Various biomarkers in body fluids can be detected through well-established methods such as enzyme-linked immunosorbent assay (ELISA), liquid chromatography–mass spectrometry, radioimmunoassay, protein microarrays, flow cytometry, etc. However, the limitations of such sensing platforms are the difficulty in real time and continuous monitoring of biomarkers. Considering the design of wearable biosensors, such detection systems are also incapable of mounting on body parts. Furthermore, these techniques use large sample volume and time-consuming experimental procedures with expensive instrumentation setups. Considering the detection of biomarkers, especially signature molecules that are a sign of chronic diseases and symptoms, the biomarkers need to be monitored continuously. Moreover, the sensor platforms also exhibit the ability to continuously monitor the concentration changes of the molecules in real time. Sweat-based stress biomarker detection therefore requires a miniaturized biointerface platform with sensor probes to continuously monitor the target analyte in real time.

Currently, the design of wearable, portable, or disposable biosensors uses plastic or paper as the electrode platform due to their light weight, flexibility, and ease of mounting to body parts in conjugation with modern communication devices.^48−50^ However, the fabrication of such wearable sensors proceeds through laser patterning, chemical vapor deposition of metal ions, lithography techniques, sputtering, spray coating, atomic layer deposition, etc. These fabrication techniques generally require complex and expensive instrumentation setups with hands-on training and skilled persons. Therefore, mass production and easy fabrication of the electrode platforms from easily available, inexpensive, and flexible materials with reduced cost are critical for the development of wearable biosensors. Taking this into account, we have designed a flexible overhead projector (OHP) sheet nanoelectrode platform through a hybrid polymerization technique consisting of an interfacial polymerization of pyrrole and electrochemical polymerization of EDOT derivatives. The novel OHP sheet electrode platform with polypyrrole and a thin layer of poly(EDOT-OH) was decorated with poly(EDOT-COOH-*co*-EDOT-EG3) nanostructures through a template-free electropolymerization method. The newly designed OHP flexible nanoelectrode platform was used for sweat NPY detection. We are unaware of any previous reports on the engineering of functionalized PEDOT nanostructures on a flexible OHP sheet to design nanobiosensor platforms with a focus on wearable electronics applications.

**Figure 1:**
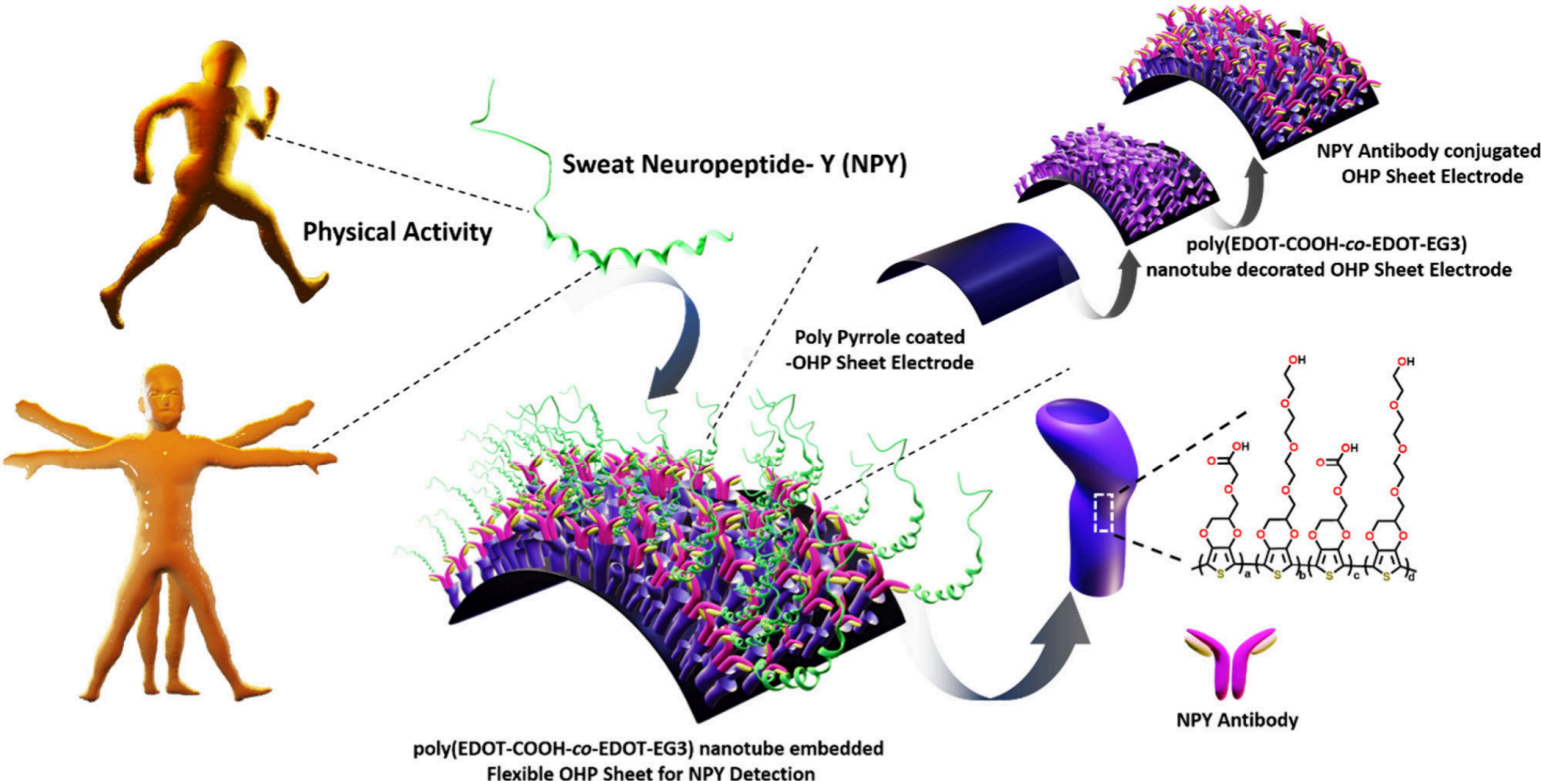
Schematic representation of an OHP sheet modified with a flexible nanobiosensor for sweat NPY detection. The nonconductive OHP sheet was coated with polypyrrole through interfacial polymerization, and poly(EDOT-COOH-*co*-EDOT-EG3) nanotubes were decorated through template-free electropolymerization. NPY-Ab was then conjugated on the polymer nanotube surface through EDC/Sulfo-NHS coupling chemistry.

Therefore, in this paper, we have developed an inexpensive and easy fabrication method for the design of nanoelectrode platforms for sweat NPY detection (Figure 1) using easily available cost-effective OHP sheets. Our method for the design of a nanoelectrode platform highlights the use of a thin, flexible, inexpensive, and easily available nonconductive OHP sheet. The nonconductive OHP sheet was converted into a conductive nanoelectrode sensor platform through the exploration of hybrid polymerization techniques involving an interfacial polymerization of pyrrole to deposit poly(pyrrole) and template-free electrochemical polymerization of EDOT-COOH-*co*-EDOT-EG3 monomers to engineer corresponding polymer nanotubes to enhance sensor sensitivity. The molecular design of EDOT-COOH offers NPY antibody (NPY-Ab) conjugation through EDC/Sulfo-NHS coupling chemistry, and triethylene glycol (EG3)-appended EDOT serves as an antifouling agent to reduce the nonspecific binding on the sensor platform. A thin layer of poly(EDOT-OH) was electropolymerized on the surface prior to polymer nanotube engineering on the electrode platform in order to enhance the attachment of poly(EDOT-COOH-*co*-EDOT-EG3). Before NPY-Ab conjugation, we optimized the electrode surface morphology by varying the electropolymerization conditions while depositing poly(EDOT-COOH-*co*-EDOT-EG3). Quartz crystal microbalance (QCM) techniques were used to observe the real-time conjugation of NPY-Ab on the polymer surface. Chronoamperometry, Fourier transform infrared spectroscopy (FTIR), and X-ray photoelectron spectroscopy (XPS) techniques also confirmed the successful immobilization of NPY-Ab on the polymer surface. Following the successful conjugation of NPY-Ab, the OHP nanosensor platform was used to detect NPY in phosphate-buffered saline (PBS) buffer (1×, pH = 7.4) with a wide linear range of concentrations from 1 pg/mL to 1 μg/mL using the chronoamperometry technique. Our newly developed OHP nanoelectrode showed a limit of detection (LOD) of 0.68 pg/mL with good linearity (*R*^2^ = 0.9841). The electrode platform exhibited superior selectivity to NPY analyte among other interfering compounds such as tumor necrosis factor α (TNF-α), cortisol, and Interleukin-6 (IL-6) in PBS buffer. Furthermore, the OHP nanosensor platform showed excellent stability, reproducibility, repeatability, and a shelf-life of 13 days. The clinical practicality of the sensor platform was confirmed by monitoring the chronoamperometric current changes when artificial sweat spiked with NPY was introduced to the sensor system. These results highlight that the newly developed OHP nanoelectrode platform was suitable for clinical applications to integrate the sensor into wearable electronics.

## Experimental Section

Monomers EDOT-COOH and EDOT-EG3 were synthesized according to our previous report, which is depicted in Scheme S1. Details of all of the reagents and materials used are provided in the Supporting Information.

### Flexible Electrode Fabrication from an OHP Sheet through Interfacial Polymerization

We used an OHP sheet available from a local market to engineer a flexible nanoelectrode platform. The size of the available OHP sheets was A4 (21 cm width × 15 cm height) with a thickness of 1 μm. The sheets were first cut into a size of 8 cm × 7 cm to keep inside a Petri dish for interfacial polymerization. Prior to interfacial polymerization, the OHP sheets were cleaned well with deionized (DI) water and ethanol for 5 min under ultrasonication, followed by plasma cleaning for 10 s. Then the OHP sheets were thoroughly dried in air. We followed the interfacial polymerization protocol from previously reported procedures with modifications.^51^ Interfacial polymerizations was performed using two immiscible solutions, such as an aqueous layer and an organic layer consisting of an oxidant and monomer solution. An aqueous solution of 0.4 M FeCl_3_·6H_2_O , and 0.4 M *p*-toluenesulfonic acid monohydrate (PTSA) was prepared separately. The two solutions were added to the OHP sheet kept previously in the Petri dish at 4 °C. Then pyrrole monomer in an organic solvent was added dropwise to the reaction mixture. The polymerization continued for 24 h. Then the electrode was washed thoroughly with methanol and DI water.

### Nanostructure Engineering on an OHP Sheet through Template-Free Electropolymerization

Flexible OHP sheets coated with polypyrrole were subjected to template-free electropolymerization using copolymers of EDOT-OH, EDOT-COOH, and EDOT-EG3. We used a potentiostat (PGSTAT128N, Autolab from Metrohm Inc.) to perform the electropolymerization process. We performed two electropolymerization processes separately as detailed below.

### Electropolymerization of EDOT-OH on a Polypyrrole-Coated OHP Sheet

The OHP sheet obtained after polypyrrole coating was cut into 1 cm × 1 cm size for all electrochemistry experiments. A kapton tape (polyimide) was adhered on one side of the OHP sheet in order to maintain identical electrode fabrication for different measurements. A typical three-electrode system was used for the electropolymerization process. For the first electropolymerization protocol, 10 mM EDOT-OH was dissolved in dichloromethane with tetrabutylammonium perchlorate (TBAP) as the supporting electrolyte. An applied constant potential of +1.2 V (vs Ag/Ag^+^) for 10 s at room temperature was used to deposit the polymer on the electrode surface. All of the monomer solutions were deaerated under a N_2_ flow prior to all of the electropolymerization experiments to avoid any effects of O_2_.

### Nanostructure Engineering of EDOT-COOH-*co*-EDOT-EG3 on the OHP Electrode

The nanotubular poly(EDOT-COOH-*co*-EDOT-EG3) top layer on the OHP sheet was constructed by template-free electropolymerization at different conditions. For that, a monomer solution of EDOT-COOH (5 mM) and EDOT-EG3 (5 mM) in CH_2_Cl_2_ containing TBAP (100 mM) was prepared. Then an applied constant potential of +1.2 V (vs Ag/Ag^+^) for 90 s at 0–2 °C was used to deposit polymer nanostructures on the OHP sheet electrode. We set a constant charge (*q*) while performing all electropolymerization experiments in order to engineer identical OHP sheet electrodes. After polymerization, the electrodes were washed with acetonitrile and double-distilled DI water to remove any unreactive supporting electrolyte and unpolymerized monomers.

### Antibody Conjugation on the Active Surface of an OHP Nanoelectrode

An OHP nanoelectrode with an under layer of polypyrrole, poly(EDOT-OH), and an upper layer of nanostructured poly(EDOT-COOH-*co*-EDOT-EG3) was modified with NPY-Ab through EDC/Sulfo-NHS coupling chemistry. In short, the electrode was treated with 0.4 M EDC and 0.1 M Sulfo-NHS in PBS (1×, pH 7.4) for 6 h to activate the carboxylic acid present on the electrode surface. After activation, the electrode surface was washed with the same buffer solution to remove any unreacted EDC/Sulfo-NHS reagents. NPY-Ab dissolved in PBS buffer (100 μg/mL) was drop-casted onto the electrode surface for 4 h at 4 °C to form amide bonds between the carboxylic acids from the top polymer layer, poly(EDOT-COOH-*co*-EDOT-EG3), on the electrode surfaces and amine groups from the antibody. Once the conjugation process was completed, the ready-to-use system was washed with PBS buffer to remove unreactive reagents from the electrode surface. The air-dried electrodes were used for the next step to detect the analyte through the chronoamperometry technique.

### Electrochemical Detection of NPY by a Poly(EDOT-COOH-*co*-EDOT-EG3)-Modified OHP Nanoelectrode

We used chronoamperometry as the electrochemical detection technique to study the interaction of NPY analyte on the newly developed OHP nanoelectrode. We measured the current response over time (*I*–*t*) when different concentrations of NPY analyte were subjected to interaction with the electrode. Prior to all experiments, the electrodes were treated with NPY analyte in PBS for 15 min to ensure antigen–antibody interaction. The electrodes were then washed in PBS buffer before each experiment. We applied 0.6 V (vs Ag/AgCl) over 100 s to measure the current while doing chronoamperometry. Each experiment was repeated a minimum of 10 times to ensure the reproducibility of the results. We prepared NPY solutions having concentrations ranging from 1 pg/mL to 1 μg/mL.

## Results and Discussion

### Synthesis of Monomers and OHP Nanoelectrode Fabrication

**Figure 2:**
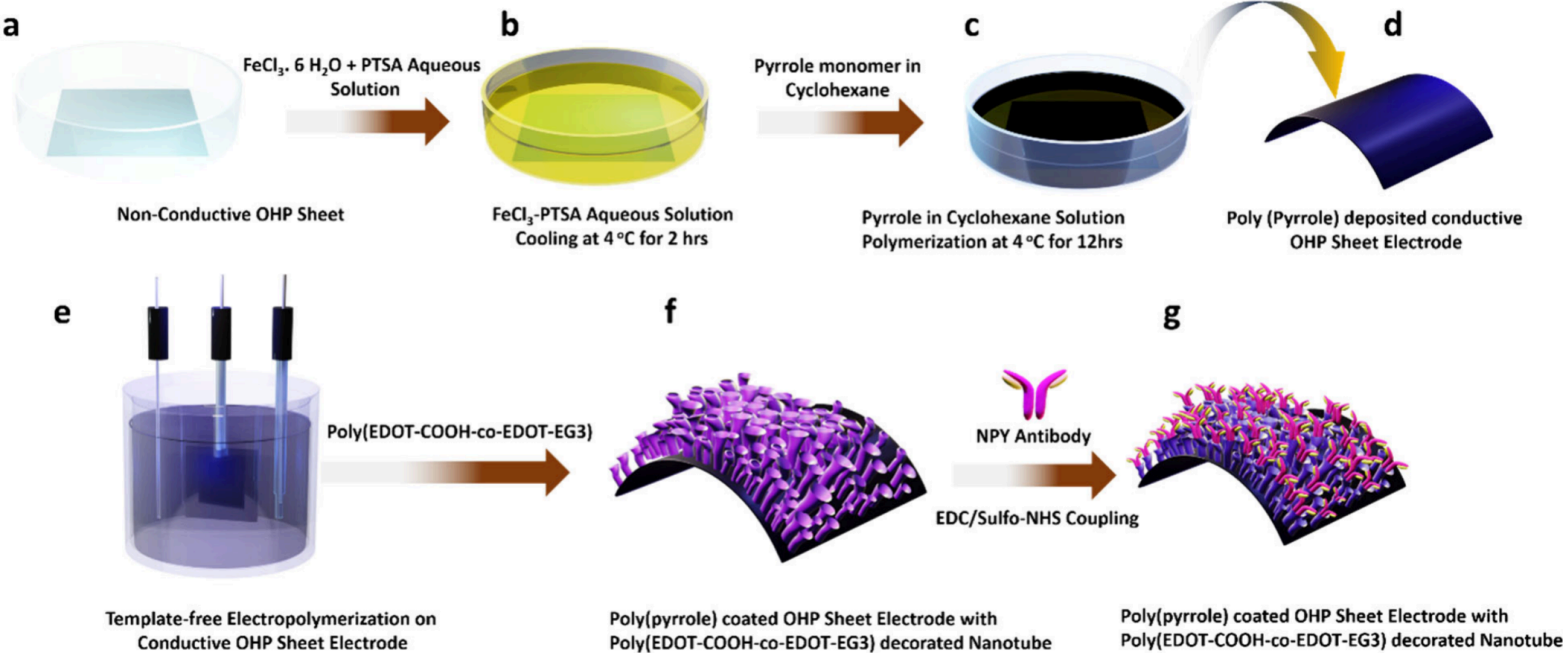
Interfacial polymerization of pyrrole (a–d) and template-free electrochemical polymerization of EDOT-COOH-*co*-EDOT-EG3 monomers on a nonconductive OHP sheet to immobilize NPY-Ab (e–g). NPY-Ab was immobilized on the OHP sheet electrode surface through EDC/Sulfo-NHS coupling chemistry.

The synthetic route for monomers can be found in the Supporting Information. In this work, we highlight the simple and inexpensive strategy to construct a nanobiosensor electrode platform from cost-effective and easily available OHP sheets. The engineering of our OHP nanoplatform consists of hybrid polymerization techniques involving interfacial polymerization of pyrrole and template-free electropolymerization of EDOT-COOH-*co*-EDOT-EG3 monomers. Our method of cost-effective engineering of an OHP-sheet-based nanobiosensor for wearable electronics consists of a very simple fabrication process without accessing highly sophisticated instrumentation beyond the clean room process and tedious design strategies as well as without any harsh chemical reactions. The complete steps involved in the hybrid polymerization technique for the electrode fabrication are illustrated in Figure 2. Our goal was to engineer a nanoelectrode with an inexpensive fabrication strategy for the wearable electronics application in mind. We selected a nonconductive OHP sheet to engineer a nanoelectrode mainly for two reasons. One is the easy availability of the material with very low cost. The other is that OHP sheets are very thin sheets with high flexibility, which is an essential feature for the construction of wearable electrodes for bioelectronics application. We commenced the engineering of an OHP nanoelectrode with interfacial polymerization. The interfacial polymerization protocol we used here was adopted from a previously reported procedure with modification as mentioned in the Experimental Section. As shown in Figures 2a and S2a, the cleaned OHP sheet with 8 cm × 7 cm dimensions was kept in a Petri dish to start the interfacial polymerization process. Our initial objective was to convert a nonconductive OHP sheet into a flexible conductive electrode platform by depositing polypyrrole material. For the oxidation of a pyrrole monomer, we used an FeCl_3_–PTSA mixture in a 1:0.8 volume ratio as the oxidant solution (Figures 2b and S2b). Following that, we observed an immediate polymerization of the pyrrole monomer when the monomer solution in a cyclohexane solvent was added dropwise to the oxidant solution, and the solution color changed from pale yellow to dark brown and then to black (Figures 2c and S2c). We confirmed that the polymerization taking place at the interface of the immiscible aqueous phase and cyclohexane organic phase was successful when the transparent OHP sheet turned black due to the deposition of polypyrrole (Figures 2d and S2d). After the interfacial polymerization of polypyrrole on the OHP sheet, we observed that the polypyrrole deposited on the OHP sheet was not uniform. Therefore, we repeated the same polymerization process one more time. Finally, we successfully obtained polypyrrole uniformly coated on the OHP sheet electrode.

**Figure 3:**
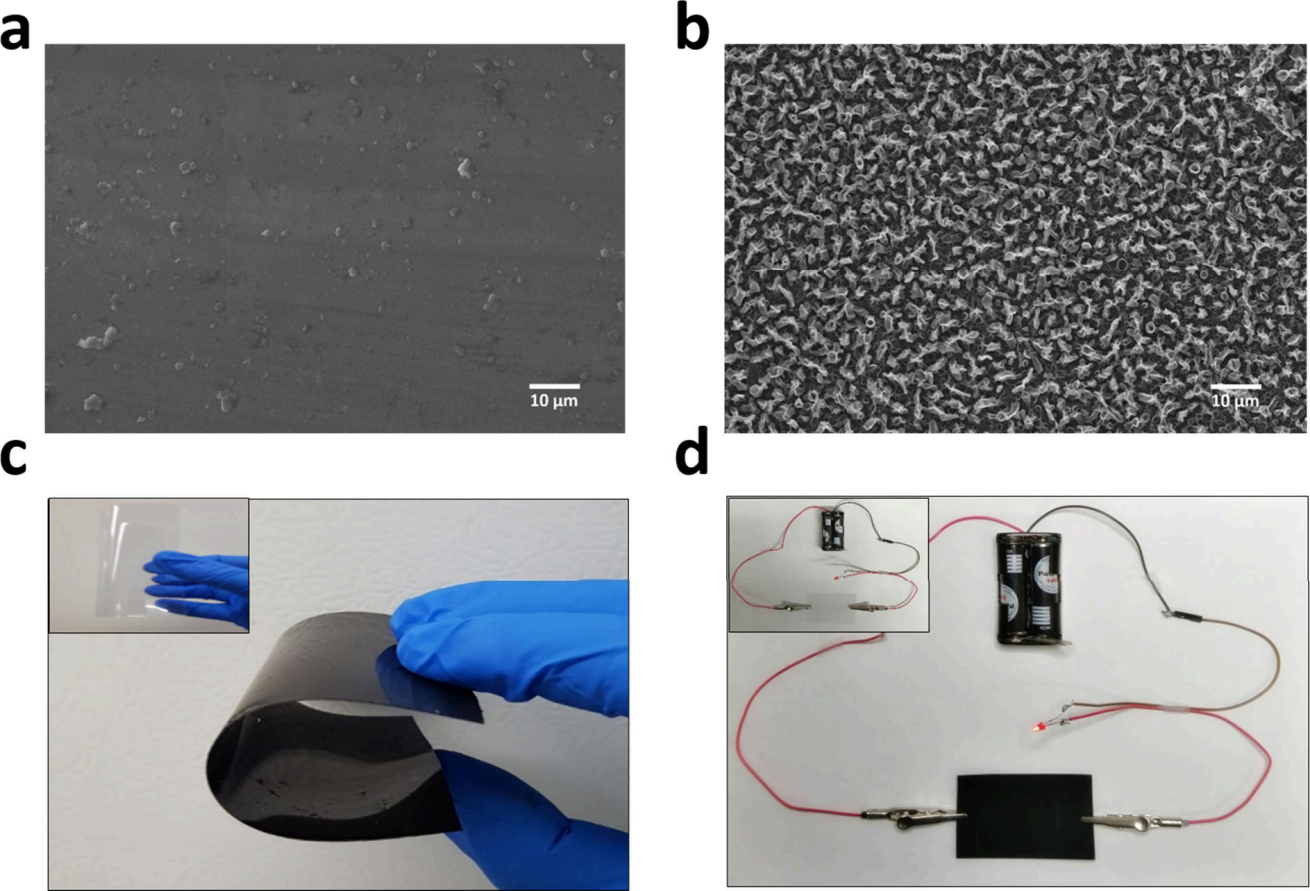
Surface morphology and electrical conductivity studies of a newly designed OHP nanosensor electrode. (a) SEM image showing a polypyrrole-coated OHP sheet. (b) SEM image highlighting poly(EDOT-COOH-*co*-EDOT-EG3) nanotubes successfully decorated on a polypyrrole-deposited OHP electrode. (c) OHP sheet before (inset) and after polypyrrole deposition. (d) Demonstration of nonconductive (inset) and conductive OHP sheets after interfacial polymerization of pyrrole.

Figure 3c highlights the successful deposition of polypyrrole on a nonconductive OHP sheet, with easy flexibility without any significant material rupturing (the inset in Figure 3c shows the bare OHP sheet). Furthermore, we tested the electrical conductivity of the OHP sheet platform before and after polypyrrole deposition. Figure 3d demonstrates the successful light-emitting diode (LED) bulb glow with a complete circuit having a polypyrrole-coated OHP sheet, while the inset figure shows that the nonconductive OHP sheet prevents the circuit completion to glow the LED bulb. Again we ensured the conductivity of the polypyrrole-coated OHP sheet using a multimeter device, as shown in Figure S3. It was confirmed that the OHP sheet did not have conductivity properties before interfacial polymerization of pyrrole (Figure S3a), while the OHP sheet turned into a conductive platform after the successful polypyrrole deposition (Figure S3b).

**Figure 4:**
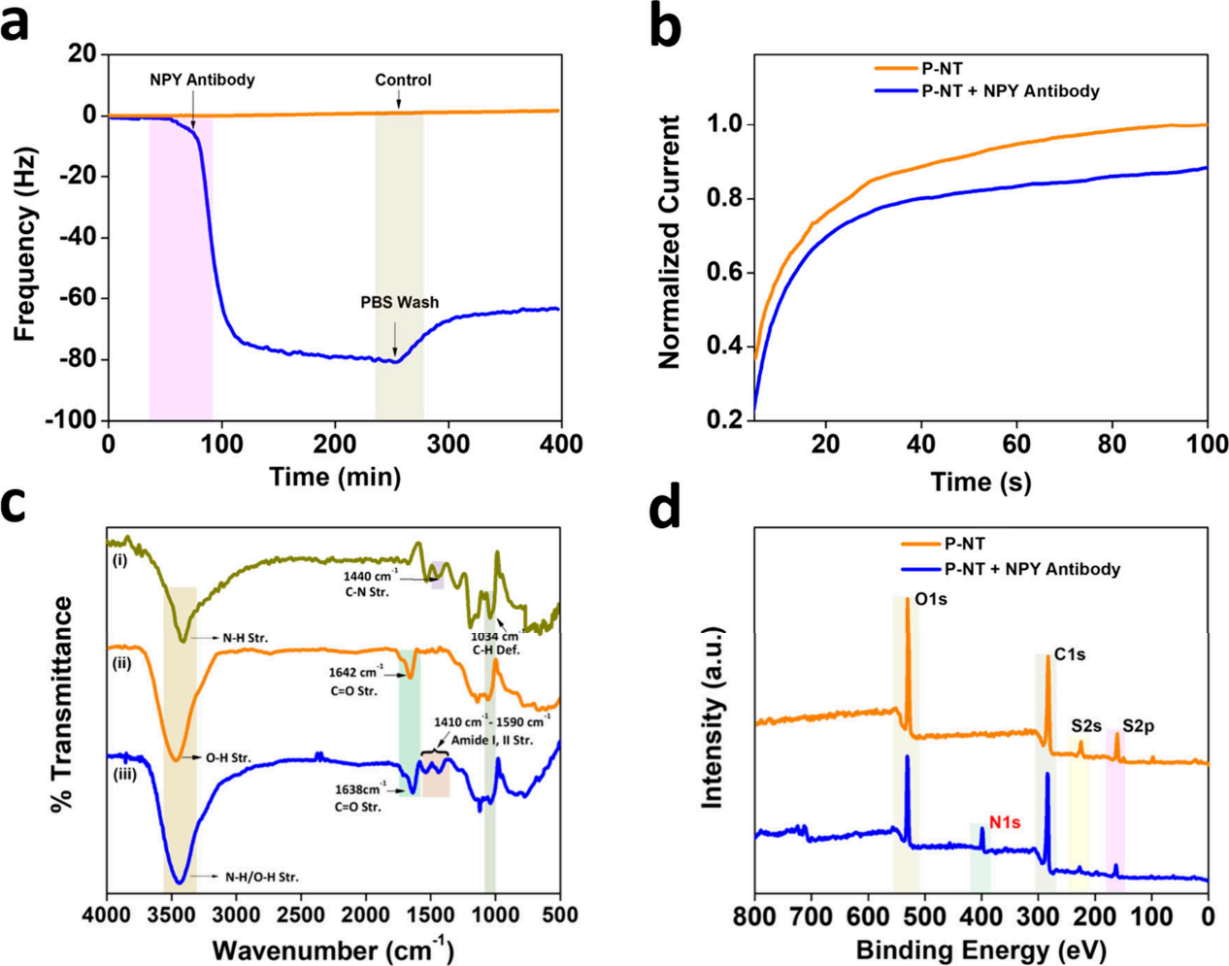
(a) Real-time monitoring of NPY-Ab binding on the P-NT surface through QCM. The drop in frequency showed the covalent attachment of NPY-Ab on the polymer surface. (b) Current response before and after NPY was immobilized on the P-NT surface. (c) FTIR spectra of (i) polypyrrole, (ii) poly(EDOT-COOH-*co*-EDOT-EG3), and (iii) a NPY-modified polymer surface. (d) XPS spectra of the P-NT surface before and after NPY-Ab conjugation.

The polypyrrole-coated OHP electrode was characterized by FTIR spectroscopy and scanning electron microscopy (SEM) techniques. Figure 4c(i) shows the FTIR spectra of polypyrrole on the electrode surface. The characteristic −C–N stretching vibrations of the polypyrrole chain at 1440 cm^–1^, C–H deformations at 1034 cm^–1^, and −N–H stretching vibrations at 3404 cm^–1^ were observed. These observations are in accordance with previous reports and thereby confirm the successful deposition of polypyrrole on the electrode surface. The surface morphology of the polypyrrole-coated OHP electrode was studied using a SEM technique. As shown in Figure 3a, the SEM image highlights that the polypyrrole-coated OHP sheet electrode did not show any significant morphology. Furthermore, the surface was not smooth and did not highlight any nanostructures. On the other hand, we observed a few bumpy and scattered morphologies on the polymer surface.

Engineering nanostructures on the OHP electrode was our main objective to design a novel flexible biosensor platform. The reason to construct such nanostructures on the electrode platform mainly highlights the larger surface area from the polymeric morphology,^20,25,33^ which can enhance the binding of NPY antibodies to ensure greater sensing of NPY analyte samples (Figure 2e–g). To engineer polymeric nanostructures on the OHP sheet electrode, template-free electropolymerization was the best choice we opted for tuning of the surface morphology of the electrode platform through control of the polymerization parameters, it is easy to perform the experiment, and it requires much less time and amount of monomers without any sophisticated instrumentation setups and tedious fabrication processes.

From our previous reports, it was observed that poly(EDOT-OH) deposition on conducting surfaces can enhance the addition of other PEDOT derivatives, particularly with a polar group appended on the polymeric side chain.^21,52^ Therefore, prior to the engineering of poly(EDOT-COOH-*co*-EG3) nanostructures on the electrode surfaces, a thin layer of poly(EDOT-OH) was deposited through electropolymerization onto a polypyrrole-coated OHP sheet. After that, template-free electropolymerization started with EDOT-COOH-*co*-EDOT-EG3 in a 1:1 ratio. In order to construct homogeneous and dense nanostructures on the electrode surfaces, we optimized the formation of nanostructures on the electrode surfaces by controlling the electropolymerization parameters (e.g., applied potential and time).

SEM images of the polymer morphology on the electrode surfaces at different polymerization conditions are shown in Figure S1. We initiated electropolymerization with a constant potential of 1.1 V (vs Ag/Ag^+^) for time durations of 30, 60, and 90 s. When the applied potential was 1.1 V and a time of 30 s, we could observe very few nanotube structures on the electrode surface (Figure S1a). When the polymerization time was increased from 30 to 60 s and then 90 s, we observed the formation of more nanotubes on the surface of the electrode (parts b and c of Figure S1, respectively). However, the SEM images showed that the nanotubes formed on the surface were irregular, less dense, and nonhomogeneous. Therefore, we increased the applied potential from 1.1 to 1.2 V with polymerization times of 30, 60, and 90 s. As shown in Figure S1d, many regular nanotube structures were observed on the electrode surface at 1.2 V and 30 s. However, the nanotubes were not dense enough to completely form on the surface. When the polymerization time was increased to 60 s, we observed more nanotubes on the electrode surface with high density. However, we observed nonhomogeneous and irregular nanostructure formation on the surface (Figure S1e).

Therefore, we increased the polymerization time again to reach 90 s from 60 s. Figure S1f shows that regular, highly dense, and homogeneous nanotubes were formed on the electrode surfaces compared to those formed at 1.2 V and 60 s duration. Out of curiosity, we also tried 1.4 V applied potential to observe polymer nanotubes on the electrode surface. However, our attempt failed because the polymer film formed at 1.4 V and 30 s duration showed many cracks on the surface and tend to slowly peel off from the surface with washing. This might be due to the higher amount of polymer film formed on the electrode surface when more polymer was deposited on the surface at a high applied potential. From the observations on the surface morphology studies, we concluded that electropolymerization at 1.2 V and 90 s could produce highly dense, regular, and homogeneous poly(EDOT-COOH-*co*-EDOT-EG3) nanotubes on the OHP electrode. (Herein, the ready-to-use OHP electrode with polypyrrole, poly(EDOT-OH), and nanotubes of poly(EDOT-COOH-*co*-EDOT-EG3) are termed “P-NT”).

The successful deposition of polymer on the electrode surface was further characterized by XPS and FTIR. The FTIR spectra shown in Figure 4c(ii) are the characteristic −C=O stretching vibrations of the polymeric chain at 1644 cm^–1^. The broad peak at 3500 cm^–1^ highlights the −OH stretching vibrations and confirms the successful deposition of polymer on the electrode surface. All of these observations confirm again that electropolymerization led to the successful deposition of a polymer nanotube layer on the OHP sheet electrode surface. Similarly, Figure 4d shows the XPS spectra of poly(EDOT-COOH-*co*-EG3) on the electrode surface (P-NT). The characteristic peaks corresponding to the binding energies of different electrons present in the orbitals were in accordance with the previous reports.^25^ Briefly, the peak at 560 eV corresponds to the O 1s electrons from the ether groups; peaks at 220 and 180 eV represent the characteristic peaks from S 2s and S 2p electrons from CH_2_ and CH linkers, respectively.

### Modification of the Electrode Surface with NPY-Ab through EDC/Sulfo-NHS Coupling Chemistry

Bioconjugation on electrode surfaces to design immunosensors is crucial for the development of highly sensitive biosensor platforms. There are many ways in which the sensor probe can immobilize on the electrode surface, in which EDC/Sulfo-NHS coupling chemistry is one of the most promising approaches. We have successfully conjugated NPY-Ab on the OHP nanoelectrode platform through EDC/Sulfo-NHS reagents. The successful conjugation of NPY-Ab on the OHP nanoelectrode was confirmed through measurement of the current changes before and after NPY conjugation, a QCM method, as well as conventional spectroscopic techniques such as XPS and FTIR. Figure 4a shows the real-time monitoring of NPY-Ab conjugation on a QCM sensor chip. The QCM technique is a versatile tool to observe the molecular interactions at the solid–liquid interfaces in real time. The change in frequency observed during QCM measurements highlight the quantitative information on NPY-Ab immobilized on the polymer surface. For the in situ monitoring of NPY-Ab binding on the polymer nanotube surface, we first activated the carboxylic group on the polymer surface by incubating the QCM sensor chip with an EDC/Sulfo-NHS coupling reagent. The activated carboxylic acid modified polymer surface on the QCM sensor chip was subjected to interaction with a PBS buffer solution (1×, pH = 7.4). Once the resonance frequency reached equilibrium, NPY-Ab (100 μg/mL) dissolved in PBS buffer (1×, pH = 7.4) was pumped through the QCM sensor. The drop in frequency observed at a time of 70 min showed the attachment of NPY-Ab on the polymer surface. The increase in frequency followed by subsequent stabilization after washing with PBS buffer (at ∼250 min) highlights the successful covalent attachment of NPY-Ab on a carboxylic acid side chain on the polymer surface. Control experiment with the QCM sensor chip modified with the same polymer without any NPY-Ab, also shown in Figure 4a. PBS buffer was used to pump over the sensor surface, and there was no drop in frequency change due to the absence of NPY-Ab in the PBS buffer solution to interact with the carboxylic acid present on the surface.

The successful attachment of NPY-Ab on the polymer surface was further confirmed by measuring the current through a chronoamperometric method. Figure 4b shows the current measurement of the OHP nanoelectrode with P-NT surface modification in PBS buffer at an applied potential of 0.6 V (vs Ag/AgCl) for a time duration of 100 s. It was observed that the current decreased after conjugation of NPY-Ab on the surface, confirming the successful covalent attachment through carboxylic acid from the P-NT surface and amine groups from the antibody, leading to the formation of amide bonds. The decrease in current might be due to the formation of an insulating layer on the electrode surface after the binding of nonconductive NPY-Ab on the electrode surface. We also extended the experimental confirmation on the covalent conjugation of NPY-Ab on P-NT-modified electrode surfaces through XPS and FTIR spectroscopic techniques. Figure 4c(iii) shows the FTIR spectra after NPY modification on the electrode surfaces. When NPY-Ab successfully conjugated on the polymer surface, characteristic amide peaks were observed due to covalent bonding between the carboxylic acid group from the polymer surface and the amine group from NPY-Ab. Figure 4c(iii) highlights the characteristic amide I and amide II stretching vibrations at 1410 and 1590 cm^–1^, respectively. We further characterized the successful conjugation of NPY on the polymer surface through XPS. Figure 4d shows the XPS spectra of the surface before (P-NT) and after (P-NT + NPY-Ab) NPY-Ab immobilization on the P-NT surface. The newly observed characteristic peak at 400.5 eV represents N 1s electrons from the nitrogen atoms of −NHCO– bonding, highlighting the successful formation of an amide bond between the polymer surface and NPY-Ab. Moreover, at 560 eV, the peak observed corresponds to O 1s electrons from the ether group, while the peak observed at 299 eV represent C 1s electrons. In addition, S 2s and S 2p electrons from the CH_2_ and CH linkers were observed at 220 and 180 eV, respectively. All of these observations were satisfied with the previous reports.

### Analytical Performance of the OHP Nanobiosensor for NPY Detection

The detection performance of the OHP nanobiosensor was monitored by measuring the current response when different concentrations of NPY antigen bind to the OHP nanoelectrode surface at ambient conditions.

**Figure 5:**
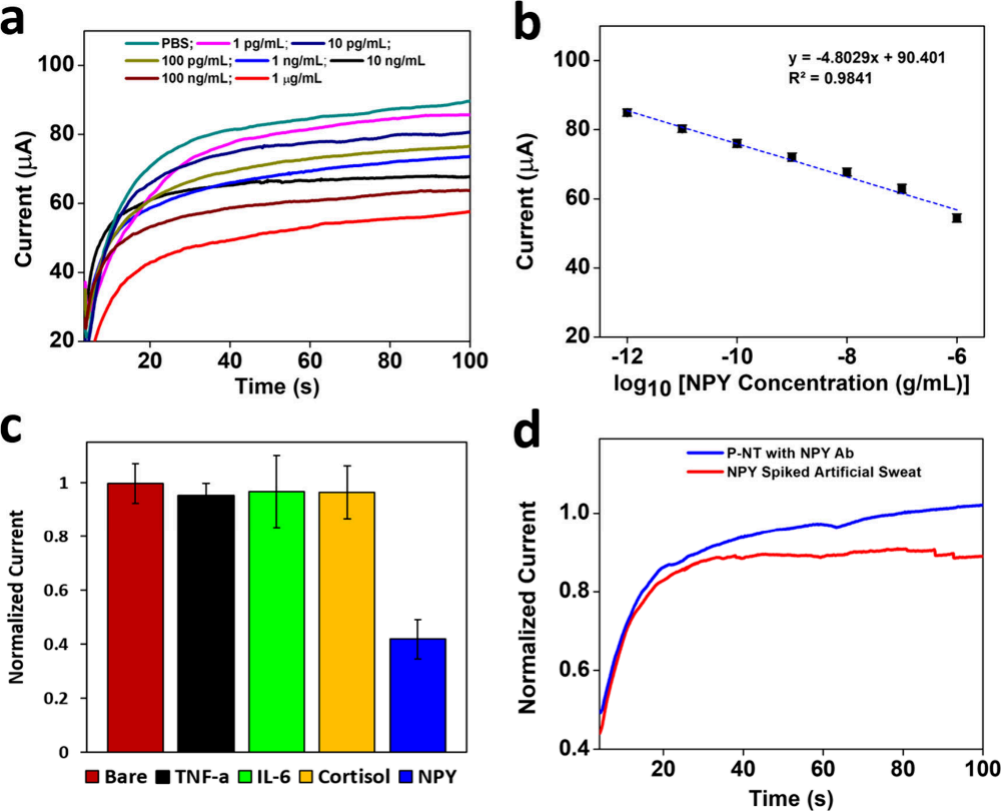
(a) Current responses for NPY detection using the OHP sheet modified with P-NT nanobiosensor. The NPY analytes added have concentrations ranging from 1 pg/mL to 1 μg/mL (PBS, 1×, pH = 7.4). (b) Corresponding calibration curve. (c) Selectivity study of the developed OHP nanobiosensor with interfering compounds such as TNF-α (1 mg/mL), cortisol (500 μg/mL), and IL-6 (1 mg/mL) in PBS. (d) Clinical practicality of the OHP nanosensor tested with artificial sweat spiked with 100 pg/mL NPY analyte.

Typically, NPY analyte interacts with NPY-Ab on the electrode surface, and the current response will be changed according to the extent of antigen–antibody interaction. Here, we used concentrations of NPY analyte ranging from 1 pg/mL to 1 μg/mL in a PBS solution (1×, pH = 7.4). Figure 5a shows the amperometric response of the current with respect to the addition of different concentrations of NPY analyte at an applied potential of 0.6 V (vs Ag/AgCl) for 100 s time duration. We prepared identical electrodes modified with 100 μg/mL antibody for detection of the NPY analyte. Each time the electrodes were treated for 15 min with different concentrations; after the desired interaction time, the electrodes were washed with the same buffer solution to remove any unbound analytes. Following that, the amperometric responses were recorded. Each experiment was repeated a minimum of 10 times in order to ensure the reproducibility of the experiments. Figure 5a highlights a distinguishable electrode response to different NPY analyte concentrations. It was observed that the current response was decreased when the concentrations of NPY were increased from 1 pg/mL to 1 μg/mL. The detection mechanism involving such a decrease in the current with an increase in the analyte concentration might be due to the formation of an insulating layer when nonconductive analyte molecules bonded strongly to the NPY-Ab immobilized on the electrode surface. The corresponding calibration curve obtained from the linear response of an amperometric current with different concentrations is shown in Figure 5b. Our OHP nanoelectrode platform exhibited good linearity (*R*^2^ = 0.9841) over a wide range of NPY analyte concentrations. The calibration curve was obtained by measuring the amperometric current response of the electrode with standard deviations having a NPY analyte concentration ranging from 1pg/mL to 1 μg/mL in PBS (1×, pH = 7.4) incubated on the electrode surface. The calibration curve can be represented by the linear regression formula *y* = −4.8029*x* + 90.401. The LOD for the sensor platform was calculated from the equation LOD = 3SD/*s*, where SD = standard deviation of the blank response and *s* = slope of the calibration curve. The LOD of the newly designed NPY nanosensor platform was calculated as 0.68 pg/mL, which is adequate to detect the minimum concentration of NPY in human sweat (5–200 pg/mL). The analytical performance of the newly developed OHP nanobiosensor for NPY detection compared to other reported NPY sensors is given in Table S1.

### Interference Effect on the Detection of NPY Analyte: Selectivity Studies

One of the major hurdles while developing biosensor platforms for healthcare monitoring is the specific detection of targeted analytes among other interfering compounds. The so-called selectivity of a biosensor platform is one of the most significant features that a biosensor can exhibit. We tested the selectivity of our newly developed NPY nanobiosensor platform in common interfering compounds such as TNF-α, IL-6, and cortisol. The normal concentrations of TNF-α, IL-6, and cortisol in sweat are 9–362 pg/mL,^53^ 5–15 pg/mL^54^ and 8–141 ng/mL,^55^ respectively. The NPY-Ab-immobilized OHP nanoelectrodes were incubated for 15 min with different solutions of TNF-α (1 mg/mL in PBS), IL-6 (1 mg/mL in PBS), cortisol (500 μg/mL in PBS), and NPY analyte (100 pg/mL in PBS). After incubation, the electrode platforms were washed with a PBS buffer solution (1×, pH = 7.4) to remove unbound compounds. Figure 5c shows the current response of the modified electrode with standard deviations (*n* = 3) at an applied potential of 0.6 V (vs Ag/AgCl) as bar diagrams. It was observed that, even though the higher concentrations of the interfering substances were incubated, the sensor platform did not display any significant changes in the current response, while the electrode incubated in a NPY solution exhibited a drop in the current, highlighting the interaction of NPY analyte with the electrode platform. These observations confirm the excellent selectivity of the nanobiosensor platform.

The clinical practicality of biosensors is important, especially those designed for wearable electronics applications. We designed our biosensor platform as a prototype for the idea of a wearable sweat nanosensor. We tested our sensor platform in NPY-spiked artificial sweat (100 pg/mL). Figure 5d shows the decrease in the current after incubation with NPY-spiked artificial sweat, confirming interaction of the sensor platform with the analyte. This result highlights that the newly designed OHP nanosensor platform can be integrated into a flexible wearable sensor platform with the objective to use it as a wearable sensor electronics system for real-time health monitoring.

### Shelf-Life, Stability, Repeatability, and Reproducibility of the NPY Nanobiosensor

**Figure 6:**
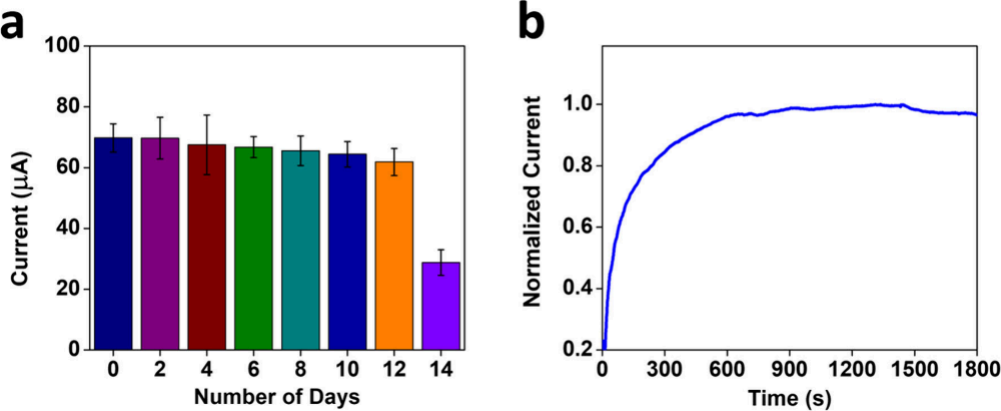
(a) Shelf-life of the OHP nanobiosensor. The bar chart represents the current–time response of the OHP nanobiosensor to 100 pg/mL NPY in PBS buffer (pH 7.4) periodically measured for 14 days. (b) Stability study of the OHP nanobiosensor represented by the current–time response for 1800 s at a potential of 0.6 V (vs Ag/AgCl) in PBS (1×, pH = 7.4).

The shelf-life study of a NPY nanobiosensor was examined by periodically measuring the amperometric current response of the NPY nanobiosensor incubated with a 100 pg/mL NPY analyte. For the shelf-life study, the electrodes were first incubated with NPY analyte for 15 min and the current response for 100 s was recorded. The electrodes were stored at 4 °C when not in use. From Figure 6a, it was observed that the OHP nanobiosensor showed a significant decrease in the current response after 13 days in PBS buffer at an applied potential of 0.6 V (vs Ag/AgCl), with 92% of the initial current response retained, showing a promising shelf-life of the newly proposed nanobiosensor. Furthermore, the long-term stability of the OHP nanosensor was investigated from the amperometric current response of the sensor platform at an applied potential of 0.6 V (vs Ag/AgCl) continuously for 1800 s time duration (Figure 6b). It was observed that the electrode platform was stable for 1800 s without a significantly large drop in the current response featuring the polymer material on the electrode surface having excellent stability without any degradation over time.

The repeatability of the newly proposed nanobiosensor was studied by the amperometric current response of electrodes interacting with the NPY analyte at three points such as low, medium, and high. The three points of different ranges in the NPY analyte concentrations were set as 1 pg/mL (low), 1 ng/mL (medium), and 1 μg/mL (high), as shown in Figure S4. Each measurement was replicated 10 times to ensure the accuracy of the measurements (*n* = 10). The different electrodes prepared identically were used to measure the current response for each concentration using the chronoamperometry method at an applied potential of 0.6 V (vs Ag/AgCl) after incubation of the electrode for 15 min in the biomarker solution in PBS (1×, pH = 7.4). We observed relative standard deviations (RSDs) of <3% for each set of testing for the three different concentration points [1 pg/mL (low), 1 ng/mL (medium), and 1 μg/mL (high)], highlighting the excellent repeatability of the sensor platform. Similarly, the reproducibility studies were conducted using five different electrode platforms, as shown in Figure S5. The identically prepared all-electrode platforms were allowed to interact with three different NPY concentrations such as 1 pg/mL (low), 1 ng/mL (medium), and 1 μg/mL (high), with each test replicated 10 times to ensure the accuracy of the experiments. The results exhibited a current response having RSDs of <3% for different NPY analyte concentrations at 1 pg/mL (low), 1 ng/mL (medium), and 1 μg/mL (high), highlighting the promising reproducibility of the sensor platform.

Our newly proposed OHP nanobiosensor for NPY detection designed from an inexpensive OHP sheet highlights promising analyte detection with excellent LOD, high stability, selectivity, repeatability, reproducibility, and clinical practicality in an artificial sweat-spiked NPY analyte. This prototype of a nanobiosensor can be integrated into a wearable bioelectronics platform for healthcare applications without a sophisticated engineering process, harsh reaction conditions and chemicals, or clean-room access or lithography techniques. We anticipate that our sensor engineering strategy highlights easy fabrication of nanobiosensors from inexpensive materials for mass production and commercialization.

## Conclusions

We have designed an OHP-sheet-based novel flexible nanobiosensor platform through hybrid polymerization techniques consisting of interfacial polymerization and template-free electropolymerization. The inexpensive and easily available nonconductive flexible OHP sheet was converted to a conductive electrode platform by depositing a layer of polypyrrole through interfacial polymerization. Nanotubes of poly(EDOT-COOH-*co*-EDOT-EG3) were decorated on the electrode platform through an electropolymerization method without using any template assemblies. We have optimized the surface morphology of the electrode platform by varying the electropolymerization conditions while depositing poly(EDOT-COOH-*co*-EDOT-EG3) nanotubes. The surface-optimized electrode platform was modified with NPY-Ab through EDC/Sulfo-NHS coupling chemistry. Our molecular design of poly(EDOT-COOH) allowed the easy immobilization of NPY-Ab, while poly(EDOT-EG3) was used as an antifouling material. QCM, FTIR, XPS, and chronoamperometry techniques were used to confirm the successful conjugation of NPY-Ab on the polymer surface. Our newly developed flexible OHP sheet nanosensor platform showed NPY detection in PBS buffer having concentrations ranging from 1 pg/mL to 1 μg/mL with an excellent LOD of 0.68 pg/mL and good linearity (*R*^2^ = 0.9841). In addition, the OHP nanobiosensor platform showed excellent stability, selectivity, repeatability, reproducibility, and shelf-life. Furthermore, we have confirmed the clinical practicality of the sensor platform by NPY-spiked artificial perspiration (100 pg/mL) through the chronoamperometric current changes, highlighting the possibility of our sensor platform to be integrated into wearable electronics for real-time health monitoring.

## Supporting Information Available

Experimental design, monomers synthesis, details of QCM measurements for real-time monitoring of NPY-Ab immobilization, stability and shelf-life studies of the OHP nanoelectrode sensor platform decorated with a poly(EDOT-COOH-*co*-EDOT-EG3) nanotube, surface morphology analysis of the OHP nanoelectrode with a poly(EDOT-COOH-*co*-EDOT-EG3) nanotube, interfacial polymerization, conductivity studies of an OHP sheet before and after polypyrrole deposition, repeatability studies and reproducibility studies of the OHP nanobiosensor electrode platform for NPY detection, and a table showing the analytical performance of the OHP nanosensor (PDF)
