# Supplementary material for: Easy-to-Engineer Flexible Nanoelectrode Sensor from an Inexpensive Overhead Projector Sheet for Sweat Neuropeptide-Y Detection
Source: ACS Appl Bio Mater. 2024 Nov 16;7(12):8423–33. doi: 10.1021/acsabm.4c01229 (PMC11653399; doi:10.1021/acsabm.4c01229)
Supplement: Supplementary file 1 — mt4c01229_si_001.pdf [file mt4c01229_si_001.pdf]

Supporting Information

Easy to Engineer Flexible Nano-Electrode Sensor from Inexpensive Overhead Projector (OHP) Sheet for Sweat Neuropeptide-Y Detection

Jayakrishnan Aerathupalathu Janardhanan[a,b,c], Jia-Wei She[a,d] and Hsiao-hua Yu*[a,b]

[a]*Smart Organic Materials Laboratory (SOML), Institute of Chemistry, Academia Sinica, Taipei City 115201, Taiwan.*

[b]*Taiwan International Graduate Program (TIGP), Sustainable Chemical Science & Technology (SCST), Academia Sinica, Taipei City 115201, Taiwan.*

[c]*Department of Applied Chemistry, National Yang Ming Chiao Tung University (NYCU), Hsinchu 300, Taiwan.*

[d]*Taiwan International Graduate Program (TIGP), Nano Science & Technology Program, Department of Engineering and System Science, National Tsing Hua University, Hsinchu 300, Taiwan.*

*e-mail: bruceyu@gate.sinica.edu.tw

Table of Contents

1. Experimental Design-Reagents and Materials- **S2**

2. Monomers-Synthesis- **S3**

3. Details of QCM Measurements for Real-Time Monitoring of Anti-Neuropeptide-Y Antibody (NPY-Ab) Immobilization- **S4**

4. Repeatability studies of OHP Nano-biosensor electrode platform for NPY Detection **S4**

5. Reproducibility studies of OHP Nano-biosensor electrode platform for NPY Detection- ***S5***

6. Stability and Shelf-Life studies of OHP nano-electrode sensor platform decorated with poly(EDOT-COOH–co–EDOT-EG3) nanotube- ***S5***

7. Surface morphology analysis of OHP nano-electrode with poly(EDOT-COOH-co-EDOT-EG3) nanotube- ***S6***

8. Interfacial polymerization- ***S7***

9. Conductivity studies of OHP sheet before and after polypyrrole deposition- ***S8***

10. Repeatability Studies ***S8***

11. Reproducibility Studies ***S9***

12. Analytical Performance of the present work compared to the previous reports on NPY detection ***S10***

13. References ***S10***

Experimental Design

Reagents and Materials.

Pyrrole (98%, Alfa Aesar), EDOT-OH (95+%; Angene Chemicals), sodium hydride (60% mineral oil), $FeCl_3.6H_2O$, sodium hydroxide (98%), Amberlite®IR-120 (hydrogen form), 1-ethyl-3-(3-dimethylaminopropyl) *N´*-ethylcarbodiimide hydrochloride (EDC-HCl, 99%), were purchased from Sigma–Aldrich. Methyl bromoacetate (98+%), sodium iodide (99+%), triethylene glycol (99%), pyridine (99%), and methanesulfonyl chloride (98%), were purchased from Alfa Aesar. Trityl chloride (98%, Acros Organics), *N*-hydroxysulfosuccinimide sodium salt (98%, Combi-Blocks), hydrochloric acid (HCl, 37%, Honeywell-Fluka™), sulfuric acid (Honeywell- Fluka™), *N*,*N*-dimethylformamide (DMF, 99.8% anhydrous, Sigma–Aldrich), tetrabutylammonium perchlorate (TBAP) from TCI Japan, were used as received. Tetrahydrofuran (THF, Macron Fine Chemicals, Avantor), and dimethylsulfoxide (DMSO, JT Baker) were purchased in sure-seal bottles, and introduced in reaction flasks under N_2 using standard vacuum/inert gas manifold techniques. Other solvents (e.g., hexane, ethyl acetate, acetonitrile, Methanol, cyclohexane) were all of analytical grade

and used as received. Elements such as carbon, oxygen, nitrogen, and sulfur elements were analyzed by A PHI-5000 VersaProbe X-ray photoelectron spectrometer. Overhead Projector sheets (OHP sheets) were purchased from a local market. Surface morphologies of the OHP nano-electrode were investigated using a Zeiss-Ultra Plus field emission scanning electron microscope operated at an accelerating voltage of 10 kV and a working distance of 10 mm. FTIR spectra were recorded using a PerkinElmer FT-IR instrument. Phosphate-buffered saline (PBS, 1x, pH 7.4, Gibco^{TM}, Thermo Fisher Scientific) was used as a standard for the QCM studies, as well as an electrolyte in the chronoamperometry measurements. Monoclonal Neuropeptide-Y antibody was purchased from Abcam. TNF-α and Interleukin-6 (IL-6) were purchased from Sino Biological. Cortisol was purchased from Sigma Aldrich. Artificial perspiration was obtained from Pickering Solutions (USA).

Monomers

Monomers were synthesized in two steps, according to the procedure displayed in Scheme S1 following the protocol we reported previously.[1]

1.Synthesis of Monomers

Synthetic route to construct EDOT-EG3 and EDOT-COOH

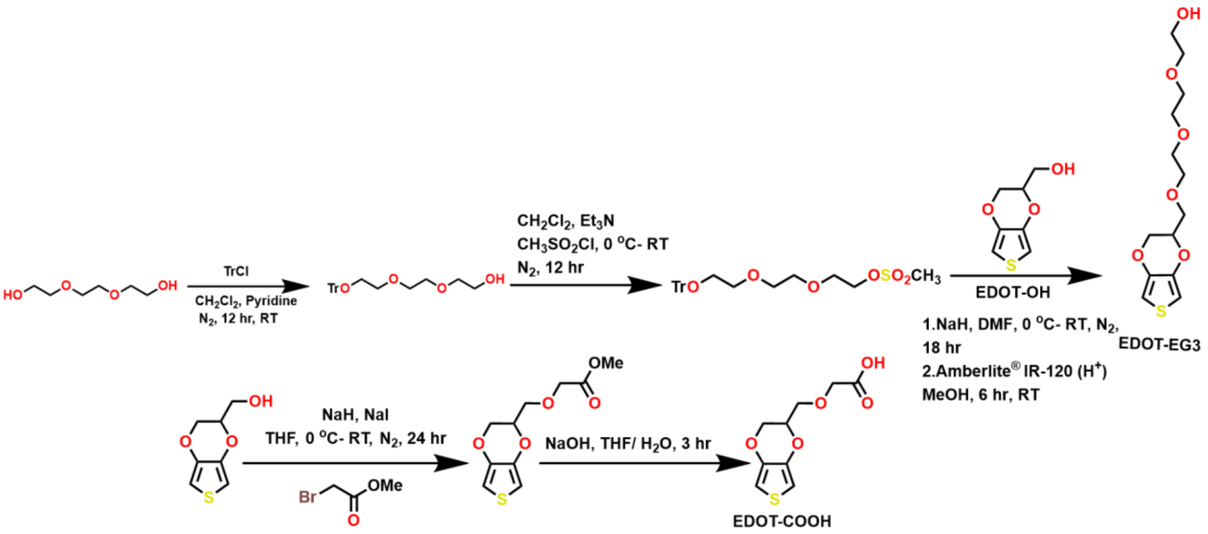

Scheme S1 Syntheses of EDOT-EG3 and EDOT-COOH

Details of QCM Measurements for Real-Time Monitoring of Anti-Neuropeptide-Y Antibody (NPY-Ab) Immobilization

Covalent conjugation of anti-Neuropeptide-Y antibody (NPY-Ab) on the polymer surface was monitored by Q-Sense E4 system (Biolin Scientific AB, QE401- F1521, Finland). Au-coated QCM sensor crystals (diameter: 14 mm) served as the working electrode to deposit poly(EDOT-COOH and EDOT-EG3) material. The sensor crystals were cleaned using piranha solution prior to the electropolymerization. Ag/Ag^{+} and Pt wire were used as reference and counter electrodes, respectively. QCM measurements were taken at a fundamental frequency of 4.95 MHz. Solutions were pumped at a flow rate of 50 μL/min using a microprocessor-controlled dispensing pump (IPC-4, Ismatec). The detailed experiments involved the activation of –COOH side chain of polymer coated QCM sensor chip through EDC/Sulfo-NHS coupling reagents. The sensor chips were then installed in the QCM chamber and PBS buffer (1×, pH 7.4) was pumped through the chamber to stabilize the fundamental frequency and overtones. Once equilibrium had been reached, a solution of anti-neuro Peptide-Y antibody in PBS buffer (100 µg/mL, PBS 1×, pH 7.4) was pumped through the chamber until the drop in frequency had stabilized. Antibody conjugation on the polymer surface was confirmed by drop in frequency after PBS washing of the polymer surface inside the chamber. QSoft 401 software was used to directly record the shift in frequency when Neuropeptide-Y antibody bonded to the polymer surface.

Repeatability studies of OHP Nano-biosensor electrode platform for NPY Detection

For the repeatability studies, we have prepared three electrodes identically for each concentrations of NPY analyte. The electrodes were produced by controlling the electropolymerization through cut-off charge to ensure equal amount of polymer deposition onto the electrode platform. The concentration of NPY antibody conjugated on the polymer surface was 100 µg/ mL in PBS solution (1 x , pH=7.4). The identically prepared NPY nano-biosensor electrode platforms were incubated with NPY biomarker at three different points of concentrations such as 1 pg/mL (low), 1 ng/mL (medium) and 1 µg/mL (high). The current response of each of these electrode platforms were measured separately using chrono amperometry technique at an applied potential of 0.6 V (vs Ag/AgCl) for 100s. The current response was measured 10 times to investigate the repeatability of the sensor platform.

Reproducibility studies of OHP Nano-biosensor electrode platform for NPY Detection

We prepared five sets of identical OHP nano-electrode sensor platform with NPY-antibody attached on the surface for reproducibility studies. In order to achieve that, we controlled the polymer nanotubes deposition by controlling the electropolymerization through cut-off charge to ensure equal amount of polymer deposition onto the electrode platform. The concentration of NPY antibody conjugated on the polymer surface was 100 µg/ mL in PBS solution (1 x , pH=7.4). Each sets of electrode platform consists of three separate identically prepared electrodes, which are used to investigate the current response for three different concentrations such as 1 pg/mL (low), 1 ng/mL (medium) and 1 µg/mL (high). The current-time response was measured for each sensor electrode at an applied potential of 0.6 V (vs Ag/AgCl) for 100s using chrono amperometry. The standard deviation and the relative standard deviation were calculated from 10 repeated measurements of current responses for each concentration points of the NPY biomarker.

Stability and Shelf-Life studies of OHP nano-electrode sensor platform decorated with poly(EDOT-COOH–*co*–EDOT-EG3) nanotube

The stability of the OHP nano-electrode sensor device was investigated by measuring current –time response at an applied potential of 0.6 V (vs Ag/AgCl) for 1800 cycles. The current response was recorded in PBS (1 x, pH = 7.4) as electrolyte solution.

The shelf-life of OHP nano-electrode sensor platform was studied by measuring current –time response at applied potential of 0.6 V (vs Ag/AgCl) when 100 pg/mL NPY analyte solution (in PBS) incubated periodically over 14 days. The sensor electrode platform was kept in refrigerator at 4 ^{o}C and periodically measured the current response with respect to time.

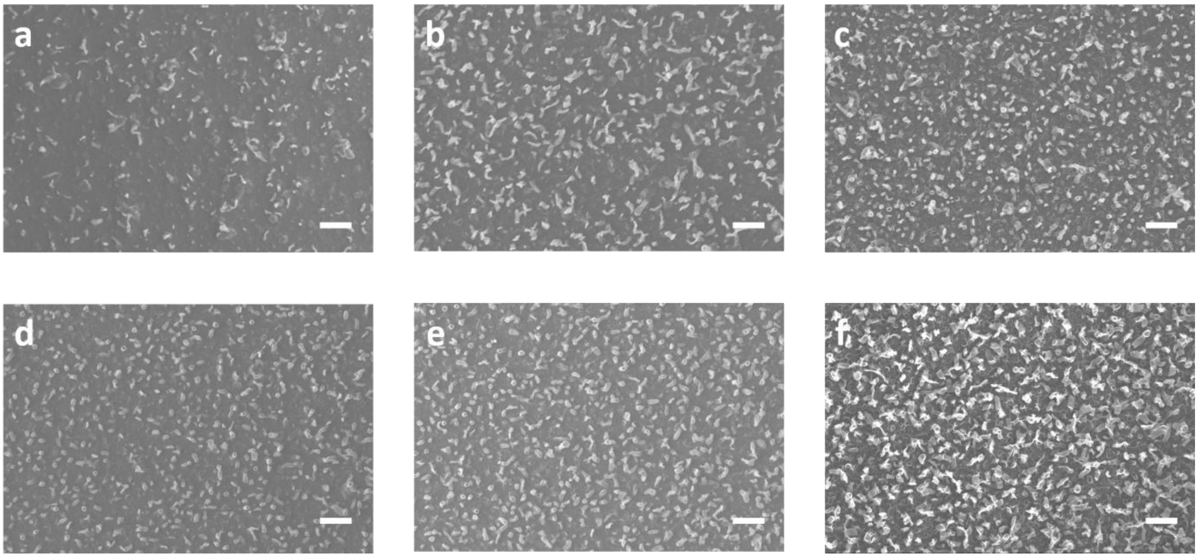

Figure S1. Scanning Electron Micrograph (SEM) images of OHP Nano-electrode platform engineered through electropolymerization of EDOT-COOH–*co*–EDOT-EG3 (1:1 feed ratio) at (a) 1.1 V, 30 s; (b) 1.1 V, 60 s; (c) 1.1 V, 90 s; (d) 1.2 V, 30 s; (e) 1.2 V, 60 s; (f) 1.2 V, 90 s (vs Ag/Ag^+ reference electrode) in CH_2Cl_2 and tetrabutylammonium perchlorate (TBAP) as the supporting electrolyte at 0- 2 °C. Scale bars: 10 µm.

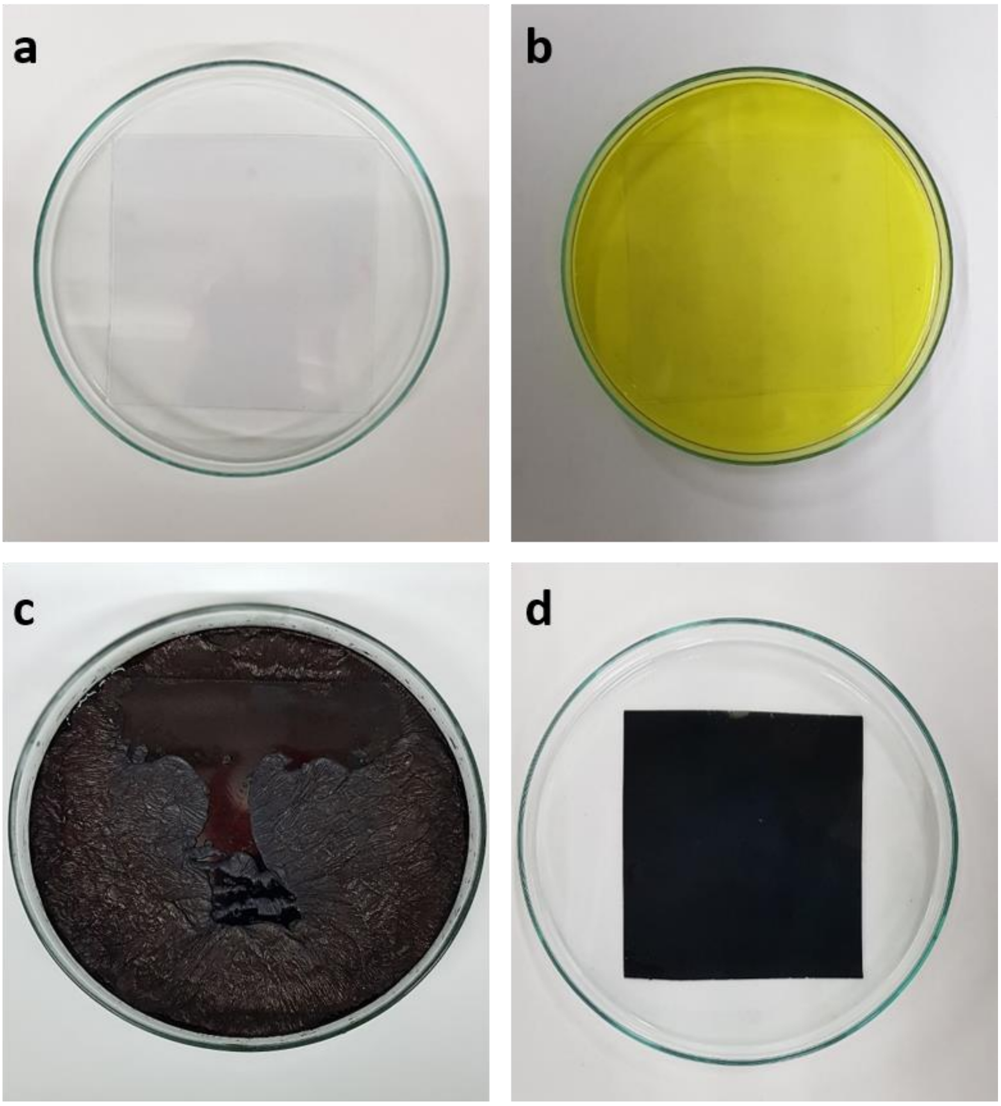

Figure S2: Process involved in the interfacial polymerization of pyrrole on non-conducting OHP sheet. (a) transparent non conducting OHP sheet in a petri dish. (b) $FeCl_3$-PTSA aqueous solution mixture added to the OHP sheet. (c) Chemical polymerization started when pyrrole monomer solution dissolved in cyclohexane added to the aqueous solution. (d) Successful coating of polypyrrole on OHP sheet.

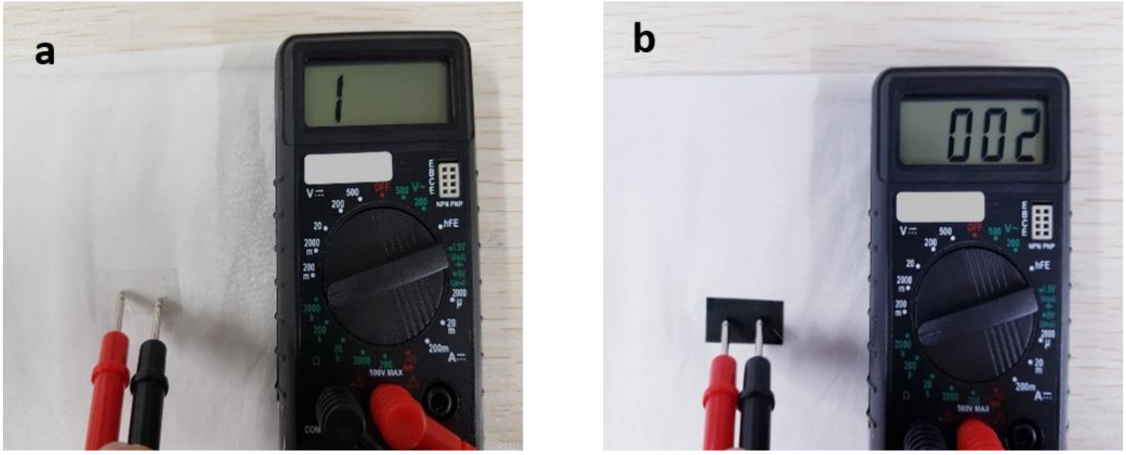

Figure S3: Successful deposition of PPy on non-conducting OHP sheet electrode. (a) non-conducting OHP sheet before PPy deposition. (b) conducting OHP sheet after PPy deposition.

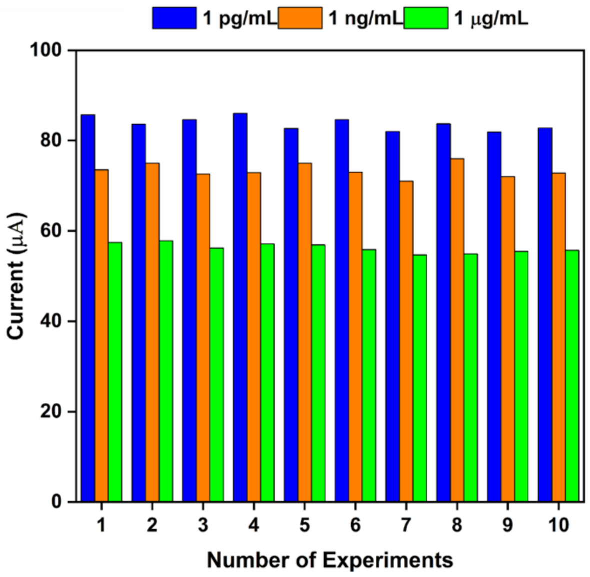

Figure S4 Repeatability studies of OHP nano-biosensor platform. The NPY antibody modified electrode platform were incubated with three different concentrations of NPY analyte such as 1 pg/mL (low), 1 ng/mL (medium) and 1 µg/mL (high). The current responses for the electrodes were measured 10 times for each concentrations using chronoamperometry technique at an applied potential of 0.6 V (vs Ag/AgCl). The relative standard deviation (RSD) calculated for each of the three concentration points of NPY biomarker were less than 3% for each studies (RSD of 1.73% for 1pg/mL, 2.08% for 1ng/mL and 1.91 % for 1 µg/mL biomarker concentrations respectively).

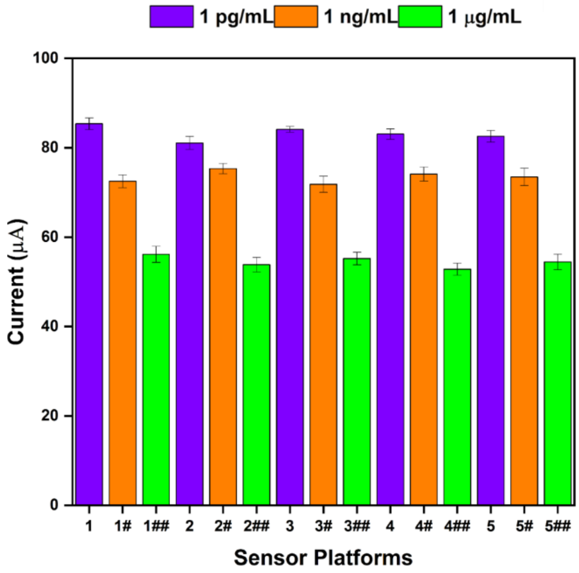

Figure S5 Reproducibility studies of OHP nano-biosensor platform using five sets of identical electrode platforms. The NPY antibody modified electrode platform were incubated with three different concentrations of NPY analyte such as 1 pg/mL (low), 1 ng/mL (medium) and 1 µg/mL (high). For each test, different electrode platforms were incubated with the NPY analyte solution. Five sets of electrodes were identically produced in which each sets have three electrode platforms named as 1, 1#, 1##, 2, 2#, 2## etc. to represent 1pg/mL, 1 ng/mL and 1 µg/mL respectively. The current response measurements were replicated 10 times for each electrode platform at these concentrations using chronoamperometry technique at an applied potential of 0.6 V (vs Ag/AgCl). The relative standard deviations (RSD) calculated for 1pg/mL = 1.94%, 1ng/mL = 1.86% and 1 µg/mL = 2.33 % biomarker concentrations respectively.

Sensor Platform	Detection technique	LOD	Linear range	Ref.
Non-porous and flexible nano-porous platform from polyamide substrate. Modified with Au	EIS, CV	10-500 pg/mL	10 pg/mL	2
submicron grating-based sensor surface coated with TiO_2-NPY Antibody	EIS	0.1 pM	0.1 pM–10 nM NPY	3
NPY Antibody-Microarray platform	CV	50 pg/mL	0.1–100 ng/mL	4
Graphene-Gold Nanocomposite-NPY aptamer		10 pM	10-1000 pM	5
carbon fiber or platinum microelectrodes-NPY Aptamer	EIS	-------------	10 to 1000 ng/mL	6
Multiplexed porous platform-NPY Antibody	EIS	--------------	50-200 pg/mL	7
OHP Sheet-PPY-Poly(EDOT-COOHco-EDOT-EG3)-NPY-Antibody	Chronoampero metry	0.68 pg/mL	1 pg/mL- 1 µg/mL	This wor k

Table S1 Analytical Performance of the present work compared to the previous reports on NPY detection

References

1. Aerathupalathu Janardhanan, J.; Chen, Y. L.; Liu, C. T.; Tseng, H. S.; Wu, P. I.; She, J. W.; Hsiao, Y. S.; Yu, H. H. Sensitive Detection of Sweat Cortisol Using an Organic Electrochemical Transistor Featuring Nanostructured Poly(3,4-Ethylenedioxythiophene) Derivatives in the Channel Layer. *Anal. Chem.* **2022**, *94*, 7584– 7593.

2. Mintah Churcher, N. K.; Upasham, S.; Rice, P.; Bhadsavle, S.; Prasad, S. Development of a Flexible, Sweat-Based Neuropeptide Y Detection Platform. *RSC Adv.* **2020**, *10*, 23173– 23186.
3. Abdallah, M. G.; Buchanan-Vega, J. A.; Lee, K. J.; Wenner, B. R.; Allen, J. W.; Allen, M. S.; Gimlin, S.; Wawro Weidanz, D.; Magnusson, R. Quantification of Neuropeptide Y with Picomolar Sensitivity Enabled by Guided-Mode Resonance Biosensors. *Sensors* **2020**, *20*, 126.
4. Jia, M.; Belyavskaya, E.; Deuster, P.; Sternberg, E. M. Development of a Sensitive Microarray Immunoassay for the Quantitative Analysis of Neuropeptide Y. *Anal. Chem.* **2012**, *84* (15), 6508– 6514.
5. Fernandez, R. E.; Sanghavi, B. J.; Farmehini, V.; Chávez, J. L.; Hagen, J.; Kelley-Loughnane, N.; Chou, C.-F.; Swami, N. S. Aptamer-functionalized graphene-gold nanocomposites for label-free detection of dielectrophoretic-enriched neuropeptide Y. *Electrochem. Commun.* **2016**, *72*, 144– 147.
6. López, L.; Hernández, N.; Reyes Morales, J.; Cruz, J.; Flores, K.; González-Amoretti, J.; Rivera, V.; Cunci, L. Measurement of neuropeptide Y using aptamer-modified microelectrodes by electrochemical impedance spectroscopy. *Anal. Chem.* **2021**, *93* (2), 973– 980.
7. Churcher, N. K. M.; Upasham, S.; Rice, P.; Greyling, C. F.; Prasad, S. Sweat Based-multiplexed Detection of NPY-Cortisol for Disease Diagnostics and Stress Management. *Electroanalysis* **2022**, *34* (2), 375– 386.
